# Supplementary material for: Induction of Stress-Induced Renal Cellular Senescence In Vitro: Impact of Mouse Strain Genetic Diversity
Source: Cells. 2021 Jun 8;10(6):1437. doi: 10.3390/cells10061437 (PMC8229707; doi:10.3390/cells10061437)
Supplement: Supplementary file 1 [file cells-10-01437-s001.zip › cells-1215286-supplementary.pdf]

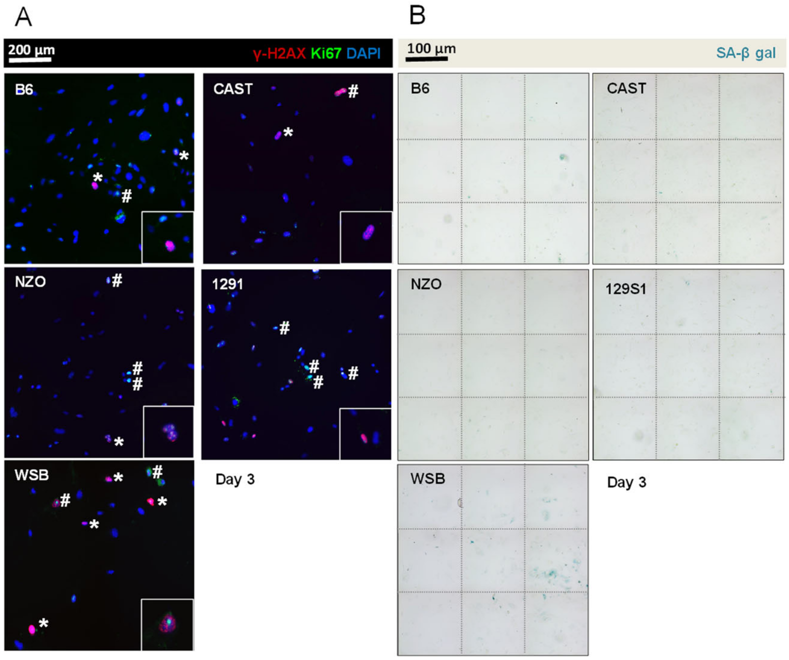


**Figure S1.** Analysis of cellular senescence at 3 days after irradiation using γH2AX and Ki67 immunofluorescence co-staining and senescence-associated beta-galactosidase (SA-β-gal). (**A**) Representative images of PTEC at day 3 after irradiation showing immunofluorescent staining for γH2AX and Ki67. Cells were counted as senescent (marked with *) when Ki67-negative and simultaneously positive for over five γH2AX foci. Ki67-positive cells are marked with #. Original magnification 400×. (**B**) Representative images of PTEC after SA-β-gal staining (positive cells are blue). Original magnification 200×.

.
